# Stimulus-Selective Response Plasticity in Primary Visual Cortex: Progress and Puzzles

**DOI:** 10.3389/fncir.2021.815554

**Published:** 2022-01-31

**Authors:** Daniel P. Montgomery, Dustin J. Hayden, Francesca A. Chaloner, Samuel F. Cooke, Mark F. Bear

**Affiliations:** ^1^Department of Brain and Cognitive Sciences, The Picower Institute for Learning and Memory, Massachusetts Institute of Technology, Cambridge, MA, United States; ^2^MRC Centre for Neurodevelopmental Disorders (CNDD), King’s College London, London, United Kingdom; ^3^Department of Basic and Clinical Neuroscience, The Maurice Wohl Clinical Neuroscience Institute, Institute of Psychiatry, Psychology and Neuroscience, King’s College London, London, United Kingdom

**Keywords:** visual recognition memory, novelty detection, primary visual cortex, cortical plasticity, experience dependent plasticity, habituation

## Abstract

Stimulus-selective response plasticity (SRP) is a robust and lasting modification of primary visual cortex (V1) that occurs in response to exposure to novel visual stimuli. It is readily observed as a pronounced increase in the magnitude of visual evoked potentials (VEPs) recorded in response to phase-reversing grating stimuli in neocortical layer 4. The expression of SRP at the individual neuron level is equally robust, but the qualities vary depending on the neuronal type and how activity is measured. This form of plasticity is highly selective for stimulus features such as stimulus orientation, spatial frequency, and contrast. Several key insights into the significance and underlying mechanisms of SRP have recently been made. First, it occurs concomitantly and shares core mechanisms with behavioral habituation, indicating that SRP reflects the formation of long-term familiarity that can support recognition of innocuous stimuli. Second, SRP does not manifest within a recording session but only emerges after an off-line period of several hours that includes sleep. Third, SRP requires not only canonical molecular mechanisms of Hebbian synaptic plasticity within V1, but also the opposing engagement of two key subclasses of cortical inhibitory neuron: the parvalbumin- and somatostatin-expressing GABAergic interneurons. Fourth, pronounced shifts in the power of cortical oscillations from high frequency (gamma) to low frequency (alpha/beta) oscillations provide respective readouts of the engagement of these inhibitory neuronal subtypes following familiarization. In this article we will discuss the implications of these findings and the outstanding questions that remain to gain a deeper understanding of this striking form of experience-dependent plasticity.

## Introduction

A defining feature of the mammalian brain is a six-layered neocortex that adapts across the lifespan to pressures within the environment as they shift (Buonomano and Merzenich, 1998; Suga and Ma, 2003). To understand this adaptive plasticity, most research has focused on the primary sensory regions of neocortex due to their proximity to sensory input and the fact that they respond to relatively unprocessed information (Mountcastle, 1997; Buonomano and Merzenich, 1998). Since the 1960s, there have been many studies investigating the profound consequences of impoverished sensory experience on the functional organization of the neocortex, particularly by studying the effects of monocular deprivation (MD) on ocular dominance (OD) in the primary visual cortex (V1) (Wiesel and Hubel, 1963; Hubel et al., 1977; Shatz and Stryker, 1978; Rittenhouse et al., 1999). The study of OD plasticity has taken advantage of the massive effects produced by deprivation to assess the capacity of the neocortex to match the altered statistics of sensory experience, the mechanisms by which those modifications occur, and the regulation of that plasticity by development and aging (Smith et al., 2009; Hooks and Chen, 2020). Additional work has been undertaken to understand the way in which supplementary experience can produce highly stimulus- and input-selective modifications of neocortical response properties in monkeys (Schoups et al., 2001) and, more recently, in mice (Gilbert et al., 2001). Although vision is not the dominant sense in mice, recent work investigating the effects of experience on the functional organization of V1 has focused on this species because of the wide array of genetic tools available for the observational and interventional approaches that reveal underlying mechanisms (Seabrook et al., 2017).

Beyond the mature research endeavor into the effects of visual deprivation, most work on visual cortical plasticity in the mouse has centered on operant conditioning, typically by making a reward contingent on the presentation of a specific visual stimulus (Andermann et al., 2010; Makino and Komiyama, 2015; Poort et al., 2015). This approach builds on the traditions of Lashley (1930) and Hebb (1949) and provides great insight into the capacity for modification of receptive fields into adulthood. However, very pronounced alterations of V1 activity can also arise simply from passive visual experience. One lasting form of plasticity, termed Stimulus-selective Response Plasticity (SRP), has been studied by multiple laboratories using a variety of stimuli and measurement techniques (Frenkel et al., 2006; Aton et al., 2014; Kaneko et al., 2017; Kissinger et al., 2018; Papanikolaou et al., 2021). SRP occurs readily through passive experience in awake mice, without requirement for a task of any kind. This has allowed for swift progress in understanding its behavioral significance and underlying mechanisms. In this review, we will discuss this progress and the surprising mechanistic complexity that has been revealed.

## Stimulus-Selective Response Plasticity

**FIGURE 1:**
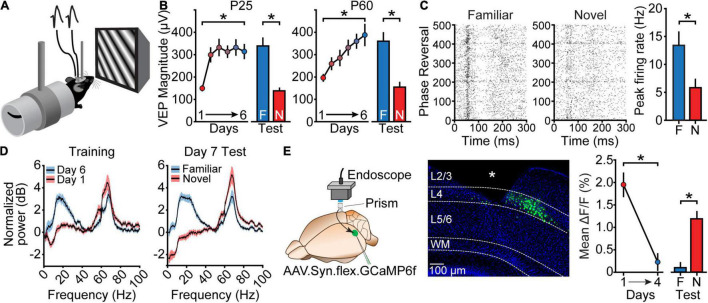
Manifestations of stimulus-selective response plasticity (SRP). **(A)** In a standard SRP experiment, mice are chronically implanted with tungsten microelectrodes in L4 of the binocular region of V1. They are then head-fixed and passively view black-and-white phase-reversing grating stimuli in order to elicit visually-evoked potentials (VEPs). **(B)** In juvenile (P25) mice, a single day of stimulus presentation leads to substantial potentiation of the VEP, which is maintained across subsequent days of viewing. The VEP elicited by a novel (N) stimulus orientation on the test day is reduced relative to the VEP elicited by the familiar (F) orientation. Adult (P60) mice require several days to reach asymptotic SRP, but after 6 days of stimulus presentation display a *F*/*N* difference similar to that of juvenile mice. Replotted from Schecter et al. (2017). **(C)** SRP also manifests as an increase in the peak firing rate of multi-unit activity recorded from L4 of V1. As with VEPs, the peak firing rate is potentiated for the familiar orientation relative to the novel orientation. Replotted from Cooke et al. (2015). **(D)** SRP is also apparent in changes in the spectral power of various oscillation frequencies. Relative to familiar stimuli, novel stimuli elicit greater high-frequency (gamma) oscillations and diminished low-frequency (alpha/beta) oscillations. This can be observed as a difference in the normalized power within these frequency ranges both when comparing the first and last days of SRP induction, and when comparing responses to familiar and novel orientations presented within the same session. Replotted from Hayden et al. (2021). **(E)** SRP manifests as a decrease in the activity of L4 principal cells when measured with calcium imaging during blocks of F stimuli. Mice expressing GCaMP6f in L4 principal cells are implanted with a prism, and a 1-p microscope is used to measure cellular fluorescence during SRP induction. Average dF/*F* responses decrease over the course of SRP induction, and are greater when viewing novel stimuli relative to familiar stimuli. White asterisk in this panel indicates location of implanted prism. Δ*F*/*F* calculated as *F*_stim_ – *F*_gray_/*F*_gray_. Replotted from Kim et al. (2020). Black asterisks throughout this figure indicate significant differences.

A convenient and time-honored method for studying visual responses in V1 is the VEP, which is usually elicited by an abrupt phase reversal of a patterned visual stimulus. Because these signals ride on ongoing fluctuations in the local field potential (LFP), they typically require time averaging of many phase reversals in a single session. The VEP reflects summed synaptic currents that vary in a stereotyped fashion as a function of latency from stimulus onset and depth in cortex. Based on the principles of current-source density analysis, a current sink at any given latency occurs at the depth with the maximum negative-going field potential (Mitzdorf, 1985). In mouse V1, the absolute maximum negative VEP is recorded in layer (L) 4, which receives the bulk of input from the visual thalamus. Because changes in this waveform are reasonably interpretable, L4 VEPs have been studied extensively to monitor experience-dependent V1 plasticity. And because VEP recordings reflect population averages, they have stability that makes them well suited for chronic recordings over weeks. SRP was an accidental discovery using this method. In experiments simply designed to track stable responses to the same stimulus —a high-contrast pattern of oriented stripes—over days, the unexpected observation was made that responses to this grating grew progressively larger, reaching an asymptotic value that could be as large as 2× the initial amplitude. By then shifting the grating to a new orientation, it was revealed that the VEP potentiation occurred only in response to the experienced stimulus (Frenkel et al., 2006) (Figures 1A,B). Subsequent studies showed that SRP is exquisitely selective for a range of stimulus properties and does not transfer to novel stimuli (Frenkel et al., 2006; Cooke and Bear, 2010). Even a slight change in stimulus orientation (of as little as 5 degrees) will elicit a significantly smaller VEP, as will changes in stimulus contrast, spatial frequency, or the eye viewing the stimulus (Frenkel et al., 2006; Cooke and Bear, 2010). While the increase in VEP magnitude elicited by a familiar visual stimulus is not observed over the course of a single recording session, pronounced potentiation manifests by the following day, suggesting a role for offline consolidation (Kim et al., 2020). In very young mice (P25), SRP can fully saturate after a single training session, but has also been found to occur in mature mice well past the critical period for other forms of visual plasticity, albeit at a slower rate of learning (Schecter et al., 2017; Eavri et al., 2018). These changes are quite stable, lasting at least weeks (Frenkel et al., 2006), and likely longer. SRP is conveniently measured using VEPs, but other approaches to monitor activity in V1 show comparable plasticity, with some very interesting variations based on the method used.

Unsurprisingly, multiunit recordings from L4 neurons reveal an increase in peak firing rate at latencies corresponding to the negative-going VEP response (Figure 1C) (Cooke et al., 2015). An increase in orientation-specific responses has also been observed in single-units from deep layers of V1 using a minor variation on the SRP induction paradigm (a single session followed by a period of complete darkness) (Aton et al., 2014; Clawson et al., 2018). Interestingly, this change in single-unit response could be prevented by disrupting corticothalamic signaling during non-REM sleep in the hours after visual experience, suggesting a potential circuit through which consolidation of SRP might occur (Durkin et al., 2017). Conversely, however, other studies that used exposure to drifting rather than phase-reversing gratings to induce and measure changes have reported a *decrease* in the average evoked single-unit firing rate in the superficial layers of V1 (Gao et al., 2021). Clearly it will be important to use a consistent stimulation paradigm to help clarify the significance of these disparate findings.

Additional readouts of SRP utilize spectral analysis of the local field potential (LFP) during visual stimulus presentation (Hayden et al., 2021). Using our induction paradigm, we have observed a striking increase in low-frequency oscillatory power during blocks of familiar stimulus viewing, particularly in the alpha- (8–12 Hz) and beta-frequency (13–30 Hz) ranges. Moreover, these increases in low-frequency oscillations are accompanied by a reduction in high gamma-frequency (65–80 Hz) oscillations (Figure 1D). As with the potentiation of the VEP and the increase in L4 peak unit activity, these oscillatory changes are reversed upon presentation of a novel stimulus (Hayden et al., 2021).

In addition to electrophysiological readouts of SRP, calcium imaging has also revealed some important aspects of SRP. Interestingly, monitoring L4 principal cell activity using one-photon calcium imaging during blocks of visual stimulation shows, unexpectedly, that activity *decreases* as a stimulus becomes more familiar across days and is reduced during familiar stimulus blocks relative to novel (Figure 1E) (Kim et al., 2020). A similar decrease has been observed in both L4 and L2/3 principal cells by another group which used 2-photon imaging to monitor cellular activity across multiple days of passive presentation of visual stimuli (Makino and Komiyama, 2015).

To summarize the constellation of findings on SRP, VEP magnitude, peak unit activity, and low-frequency oscillations within L4 of V1 all appear to increase with stimulus familiarity, while calcium responses and high-frequency oscillations within this same layer decrease. One possible way to reconcile these observations would be for familiar stimuli to elicit an increase in the *peak* unit firing of thalamo-recipient L4 principal cells (revealed by VEPs and short latency spiking), followed by decreased activity in the intervals between phase reversals (revealed by calcium imaging). Such a redistribution of activity could explain the observations described above and could be accounted for by recruitment of polysynaptic inhibition, which we discuss later.

Regardless of the underlying cellular activity reflected by LFPs and VEPs, these simple recording methods provide a relatively easy and stable means of observing a long-lasting form of plasticity over days and weeks. Moreover, this approach shows promise for translating SRP measurements into human subjects, for which VEPs and oscillations are routinely recorded non-invasively using EEG. The ease with which SRP can be induced and measured, the large effect size produced, and its exquisite stimulus-selectivity enables longitudinal experiments with repeated epochs of comparable plasticity in the same animals. This feature provides the potential for longitudinal crossover designs to test candidate treatments for dysregulated cortical plasticity in models of neurodevelopmental disorders (Cooke and Bear, 2012) or dementia (Papanikolaou et al., 2021). Recent progress in understanding the behavioral significance of SRP and identifying the key underlying mechanisms have only enhanced the potential for its translational utility.

## Orientation-Selective Habituation

The electrophysiological readouts of SRP closely mirror changes in behavior that occur over the same time course, reflecting what appears to be a fundamental form of long-term, stimulus-selective habituation. This learning effect emphasizes the significance of studying SRP given the importance of habituation as a foundational process for cognition (Schmid et al., 2014; Cooke and Ramaswami, 2020) and the strong evidence of dysregulated habituation in a number of psychiatric and neurological conditions (Ramaswami, 2014; McDiarmid et al., 2017). Habituation serves as a foundation for higher order cognition by filtering out neutral elements in the environment and prioritizing responses to novel or behaviorally salient stimuli. When it goes awry there can be devastating consequences for selective attention and energy conservation (Rankin et al., 2009; Cooke and Ramaswami, 2020). These adaptive filtration processes have been well studied over relatively short timescales using electrophysiological readouts such as mismatch negativity (Naatanen et al., 2007), but less is known about habituation over longer timescales.

**FIGURE 2:**
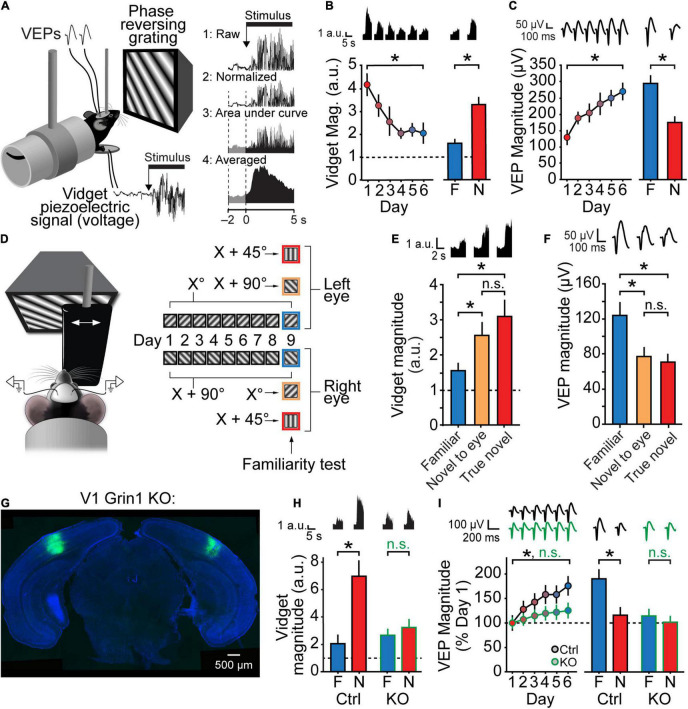
Stimulus-selective response plasticity and orientation-selective habituation (OSH) are input-specific and require NMDARs in V1. **(A)** A piezoelectric device situated beneath the forepaws of a mouse during a standard SRP experiment can be used to measure movements of the animal elicited by stimulus onset. The raw voltage recording is rectified and then normalized to the pre-stimulus onset period. The area under the curve is averaged to determine the magnitude of the visually evoked fidget (the “vidget”). **(B)** Over the course of a typical SRP experiment, the magnitude of the vidget diminishes as the animal becomes familiar with the same stimulus across days. Presentation of a novel stimulus elicits a larger vidget relative to presentation of the familiar stimulus, demonstrating the stimulus-selectivity of habituation. **(C)** Changes in the vidget follow a similar timecourse to SRP, but in the opposite direction of change observed for VEPs. **(D–F)** Both SRP and OSH are eye specific. **(D)** Distinct orientations were presented monocularly to each eye. On test day, VEPs and vidgets were measured in response to monocular presentation of the stimulus orientation familiar to the eye (blue), the orientation familiar to the opposite eye but novel to the viewing eye (yellow), and an orientation novel to both eyes (red). **(E)** Relative to the familiar stimulus for each eye, the vidget evoked by both novel stimuli were significantly increased, and the VEPs evoked by both novel stimuli were significantly decreased. There were no significant differences between the responses to the stimuli that were novel to either eye and the true novel stimuli. **(G–I)** Both SRP and OSH require NMDARs in V1. **(G)** Grin1^fl/fl^ mice were injected with a virus expressing either Cre recombinase with a GFP tag (KO) or a control virus expressing GFP alone (Ctrl). **(H)** Ctrl mice show normal OSH, but KO mice do not display a significant familiar-novel difference for vidgets. **(I)** Ctrl mice show normal SRP induction (days 1–6) and expression (day 7 test), but both are disrupted in KO mice. Figure replotted from Cooke et al. (2015). Significant comparisons are denoted throughout by an asterisk and non-significant comparisons by n.s.

When first presented with an oriented grating stimulus, mice produce an easily measured visually evoked fidgeting behavior (Figure 2A) that subsides with increasing familiarity via a process we have termed orientation-selective habituation (OSH). With a similar degree of selectivity to SRP, OSH is reversed and movement re-emerges upon presentation of a novel stimulus (Figure 2B) (Cooke et al., 2015; Kaplan et al., 2016; Fong et al., 2020; Finnie et al., 2021). VEP recordings from these head-fixed mice reveal that these behavioral changes occur along a comparable timecourse to SRP (Figure 2C). A similar pattern of habituation is observed in freely moving mice, which orient to and then actively explore these same phase-reversing, oriented, sinusoidal grating stimuli, much as seen with novel objects (Ennaceur and Delacour, 1988; Cooke and Bear, 2015). Notably, not only do mice habituate to the visual stimulus over days, but subsequent VEP recordings from these mice under head-fixation reveal the expression of SRP in response to the habituated stimulus (Cooke et al., 2015). It is tempting, therefore, to attribute the novelty-induced movement seen in head-fixed mice to an orienting response (Sokolov, 1963), but further work is required to determine the precise relationship between freely-moving and head-fixed habituation.

One concern that has recently been addressed is the degree to which the V1 phenomenology observed during SRP is actually a readout of the diminishing movement that occurs as the animal undergoes habituation. This concern arises due to studies showing movement can have profound influence over V1 neural activity (Niell and Stryker, 2010; Fu et al., 2014; Stringer et al., 2019). In the SRP paradigm, stimulus onset produces movement which diminishes with habituation, but this behavior does not persist beyond the first 10 s or so of blocks of stimulus presentations that last longer than a minute, whereas differences in the VEP and spectrums for familiar and novel stimuli persist throughout the entire stimulus block (Hayden et al., 2021). Moreover, it has recently been shown that pronounced SRP is expressed if analysis is explicitly restricted to periods of no movement (Papanikolaou et al., 2021).

Another concern is that the phenomenology observed in V1 during OSH reflects a global brain state associated with reduced arousal. This possibility is addressed by clear evidence that pupil size, which is an established readout of reduced arousal that can influence V1 activity (Vinck et al., 2015), does not change with familiar stimulus presentation and SRP using standard induction protocols (Hayden et al., 2021). Thus, SRP and accompanying shifts in the frequency composition of the LFP are clear cortical correlates of long-lasting habituation with some basis in V1. Interventional approaches have been required to understand the shared mechanisms that exists between these phenomena. We will now explore these findings.

## Stimulus-Selective Response Plasticity and Orientation-Selective Habituation Rely on the Mechanisms of Hebbian Synaptic Potentiation

In addition to their stimulus specificity, SRP and OSH share several other important features indicating that they involve similar mechanisms. Both are blocked by manipulations that target V1 (Cooke et al., 2015), indicating a cortical basis, which differentiates them from other innate visual phenomena (Douglas et al., 2005; Shang et al., 2018). Additionally, both SRP and OSH rely upon mechanisms of Hebbian synaptic strengthening, which was famously proposed as the core cortical mechanism of visual learning (Hebb, 1949). This lasting form of synaptic plasticity has typically been modeled in V1 by the experimentally induced form of plasticity known as long-term potentiation (LTP) (Kirkwood and Bear, 1994). Several lines of evidence indicate commonalities between this process and SRP:

### Input Specificity

One of the defining features of Hebbian plasticity is its input specificity. This input specificity can be beautifully demonstrated in V1 LTP using a two-pathway design (Kirkwood and Bear, 1994); stimulating a subset of inputs to a given neuron recruits molecular machinery to potentiate the synapses activated by that subset of inputs, while sparing other neighboring inputs. Similarly, presenting stimuli to only one eye during SRP induction potentiates that monocular VEP, but this potentiation does not transfer to VEPs elicited by the same stimulus through the other eye (Frenkel et al., 2006). OSH is similarly eye-specific (Figures 2D–F) (Cooke et al., 2015), as with many forms of visual perceptual learning (Gilbert et al., 2001). The stimulus selectivity of SRP could thus be explained by Hebbian potentiation occurring at a subset of thalamocortical synapses. Cells in the thalamo-recipient layers of V1 are selective for both stimulus orientation and spatial frequency of grating stimuli (Seabrook et al., 2017), so potentiation only of the synapses driven by the trained stimulus could account for the increase in the VEP specific to that stimulus.

### Requirement for NMDA Receptors in V1

The NMDA class of ionotropic glutamate receptor serves as a core mechanism of most forms of Hebbian plasticity (Collingridge and Bliss, 1995) and is critical for LTP at thalamo-cortical synapses in V1 (Heynen and Bear, 2001). The original description of SRP found that systemic injections of the NMDA receptor antagonist CPP prior to each day of training completely abolishes SRP acquisition in juvenile mice, indicating that normal function of NMDARs is critical for SRP (Frenkel et al., 2006). Of course, this alone does not indicate that NMDARs within V1 are responsible for SRP. However, more recently it was found that local infusion of the NMDAR antagonist AP5 into V1 also blocks SRP acquisition, as does genetically ablating GluN1—an obligatory subunit of the NMDA receptor—only within binocular V1. Importantly, this treatment similarly prevents OSH (Figures 2G–I) (Cooke et al., 2015).

### Requirement for Other Molecular Mechanisms Shared With Hebbian Long-Term Potentiation

The NMDA receptors are involved in many processes beyond simply mediating LTP (Iacobucci and Popescu, 2017), but their activation triggers a cascade of molecular events that are critical for synapses to be strengthened when LTP does occur. Strong NMDAR activation leads to the postsynaptic insertion of AMPA receptors, and the strengthening of these synapses is maintained by the constitutively active kinase PKM-zeta. Manipulations that disrupt any of these steps downstream of NMDAR activation also disrupt SRP. Prevention of AMPAR insertion by viral transfection of the C-terminal domain of GluR1 prevents the increase in VEP magnitude typically observed during SRP acquisition, and disrupting PKM-zeta activity by infusing the zeta inhibitor peptide (ZIP) into V1 eliminates previously consolidated SRP (Cooke and Bear, 2010) and OSH (Cooke et al., 2015).

### Mimicry and Occlusion of Thalamocortical Long-Term Potentiation

A compelling line of evidence for shared mechanism between biological processes is that they not only mimic one another but also are mutually occluding. Like SRP, inducing LTP using theta-burst stimulation (TBS) of the lateral geniculate nucleus (LGN) leads to long-lasting increases in the magnitude of L4 VEPs (Heynen and Bear, 2001; Kuo and Dringenberg, 2009; Cooke and Bear, 2010), so the two processes do mimic one another. However, the LTP effect is indiscriminate for stimulus properties and potentiates VEPs evoked by a wide range of visual stimuli. Importantly, SRP induction following TBS-induced LTP fails to increase the VEP magnitude further for the trained orientation and prevents the development of a familiar-novel difference. This occlusion effect is also bidirectional, as saturation of SRP by several repeated days of visual stimulus presentation prevents the subsequent further potentiation of the VEP by theta-burst stimulation of the thalamus (Cooke and Bear, 2010). The TBS-induced LTP effect on the VEP is, however, manifest for novel stimuli, suggesting that only those synapses already potentiated by visual experience are no longer available for further Hebbian potentiation.

Collectively, when considering these lines of evidence, the most parsimonious explanation for SRP has been that it is simply a strengthening of thalamo-cortical inputs onto L4 cells tuned to the trained stimulus. However, even at the time of discovery, there were aspects of SRP that presented confounds to this simple interpretation. While LTP in V1 manifests almost immediately after stimulation of the LGN, it can take hours for SRP to be expressed after exposure to a novel visual stimulus. Further, SRP is not only selective for orientation, but also appears to be selective for stimulus contrast. While orientation selectivity is encoded within receptive fields, contrast selectivity is not (Priebe and Ferster, 2008). The phenomenon of contrast invariance in V1 receptive fields reveals this distinction and likely reflects computations occurring in the thalamus (Palmer and Miller, 2007). Thus, if SRP reflects purely feedforward synaptic potentiation onto thalamo-recipient L4 neurons, one would expect to see potentiation of the VEP for familiar stimulus orientations regardless of stimulus contrast, but this is not in fact the case—training with a stimulus presented at 100% contrast does not elicit a familiar-novel difference when presented at 50% contrast on the test day (Cooke and Bear, 2010).

More recent studies have provided direct evidence for mechanisms of SRP that are inconsistent with a purely feedforward plasticity onto L4 principal neurons. We will now consider this evidence in order to explore alternative interpretations for the circuit-level implementation of SRP.

## Stimulus-Selective Response Plasticity is Not Simply Hebbian Potentiation of Thalamocortical Synapses in Layer 4 of V1

While there is extensive evidence that SRP involves mechanisms of Hebbian synaptic plasticity within V1, identifying the precise locus of plasticity has proven more elusive. The eye-specificity of SRP suggests that plasticity occurs prior to binocular integration. Given that within rodent binocular V1, in contrast to that of carnivores and primates, thalamo-recipient neurons exhibit binocularity (Drager, 1974), the obvious interpretation is that modifications underlying SRP occur at thalamocortical synapses. Layer 4—the site of largest change in the VEP during SRP (Cooke et al., 2015) and the canonical thalamic input layer of V1—would therefore seem to be the likeliest candidate site of plasticity. However, three lines of evidence indicate that thalamocortical synapses onto L4 glutamatergic principal neurons are not the primary locus of modification. First, VEPs driven purely by thalamocortical inputs can be isolated by blocking all local V1 spiking activity with the GABA_A_ agonist muscimol (coupled with the GABA_B_ antagonist SCH50911 to prevent non-specific binding to pre-synaptic terminals) and can thereby prevent intracortical synaptic transmission. This treatment preserves the OD shift after MD during the critical period (Khibnik et al., 2010), but eliminates the difference in the magnitude of VEPs elicited by familiar and novel stimuli after SRP, indicating a requirement for intracortical activity in the expression of SRP (Cooke and Bear, 2014). Second, the differential cortical response to familiar and novel stimuli after SRP is not observed at block onset, comprising the first stimulus presentation, but arises by the second stimulus (the first literal phase reversal), suggesting a requirement for some form of recurrent activity (Kim et al., 2020; Hayden et al., 2021). Third, knocking out the mandatory GluN1 subunit of NMDA receptors in L4 glutamatergic principal cells has no effect on SRP. While this highly selective manipulation prevents weakening of inputs from the deprived eye during MD in the critical period, it does not impair either SRP or OSH (Fong et al., 2020). As SRP is known to be disrupted by genetically or pharmacologically blocking NMDA receptors within V1, this final finding strongly indicates that NMDA receptors in some other class of cells within V1 are responsible for the potentiation of the VEP observed in SRP.

It is well established that thalamic inputs do not solely target L4 principal cells in V1. Inhibitory cells in L4 (Cruikshank et al., 2007) and principal cells in other cortical layers (Antonini et al., 1999; Beierlein and Connors, 2002; Liu et al., 2008; Constantinople and Bruno, 2013) also receive strong thalamic input. It is therefore likely that the increase in L4 responses observed over the course of SRP may in fact reflect modification of thalamocortical synapses onto other populations of neurons in V1, which in turn influences the activity of L4 principal neurons to produce SRP expression. We will now explore these candidates to consider the likeliest site of plasticity mediating SRP.

## Key Elements of Cortical Inhibitory Circuitry

To understand how inhibitory interneurons might contribute to SRP, it is necessary to understand their role in the standard circuitry of the neocortex. GABAergic interneurons make up only 10–20% of the rodent cortex (Meyer et al., 2011), but exert powerful control over cortical processes. These cells have a wide range of morphologies, gene expression patterns, and electrical properties (Rudy et al., 2011; Tremblay et al., 2016), but can broadly be grouped into three non-overlapping types: parvalbumin-positive (PV+) cells, somatostatin-positive (SOM+) cells, and serotonergic-receptor-positive (5HT3aR+) cells, the last of which includes vasoactive intestinal polypeptide-positive (VIP+) cells (Lee et al., 2010). Much of the research into the role of inhibition in cortical processing tends to focus on PV+, SOM+, and VIP+ interneurons, which collectively account for 60% of all GABAergic neurons (Lee et al., 2010; Xu et al., 2010).

PV+ interneurons are abundant across all cortical layers, with an especially high concentration in L4, the primary thalamorecipient layer (Xu et al., 2010). They are notable for their rapid firing rates and thin spikes (Kawaguchi and Kubota, 1997), and can be divided into two main subgroups: basket cells, which form dense connection with the soma and proximal dendrites of principal cells, and chandelier cells, which target the axon initial segment (Rudy et al., 2011; Harris and Mrsic-Flogel, 2013; Tremblay et al., 2016; Hooks and Chen, 2020). This connectivity poises PV+ cells to regulate the production and timing of action potentials generated by principal cells, enabling them to exert strong control over the gain and synchrony of the cortex (Tremblay et al., 2016). While most cortical PV+ cells form connections with nearby cells, there is also a class of L6 PV+ cells found in V1 that are driven by activity in L6 corticothalamic cells and modulate a column of visual cortex (Olsen et al., 2012; Bortone et al., 2014; Guo et al., 2017). These L6 PV+ cells are of great interest and will be discussed later.

Relative to PV+ cells, SOM+ inhibitory interneurons have a slower firing rate more similar to that of principal cells (Ma et al., 2010; Urban-Ciecko and Barth, 2016). SOM+ cells in the cortex are largely Martinotti-type cells that target the dendrites of principal cells, particularly more distally, affording them control over the integration of dendritic inputs onto excitatory neurons (Rudy et al., 2011; Harris and Mrsic-Flogel, 2013; Tremblay et al., 2016; Hooks and Chen, 2020). SOM+ neurons also form connections with all other classes of interneurons (Pfeffer et al., 2013), and play an important disinhibitory role in L4 by targeting and suppressing PV+ cells (Xu et al., 2013). Individual excitatory input to SOM+ cells is weak, requiring strong (or coordinated) activity to evoke activity (Urban-Ciecko and Barth, 2016).

Unlike PV+ and SOM+ cells, which tend to form promiscuous connections with nearby excitatory cells (Fino and Yuste, 2011; Packer and Yuste, 2011), VIP+ interneurons play a largely disinhibitory role. VIP+ cells are most dense in superficial cortical layers (Xu et al., 2010), where they primarily target SOM+ cells (Pfeffer et al., 2013). These neurons receive substantial top-down inputs from higher-order cortical areas and neuromodulatory inputs from subcortical nuclei, leading to substantial task- and state-dependent influence on cortical processing (Fu et al., 2014; Zhang et al., 2014; Kullander and Topolnik, 2021; Niell and Scanziani, 2021).

## Stimulus-Selective Response Plasticity Expression Involves Two Competing Populations of Inhibitory Interneurons

**FIGURE 3:**
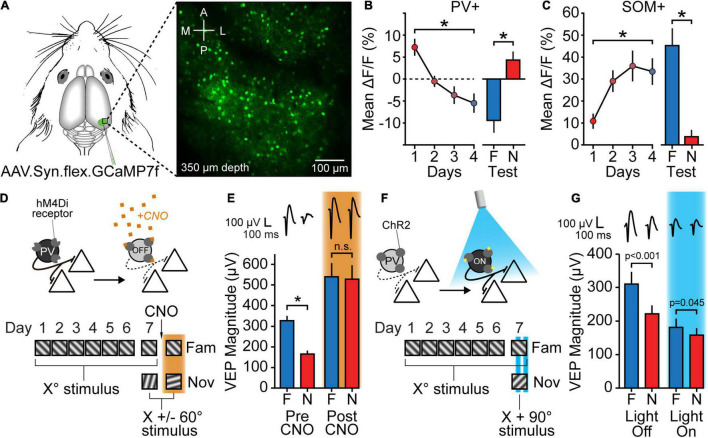
Inhibition plays a role in SRP expression. **(A)** Mice expressing Cre recombinase in either PV+ or SOM+ inhibitory neurons were injected with a Cre-dependent GCaMP7f virus in V1. Activity in PV+ or SOM+ cells in L4 was then recorded using a 2-photon microscope. **(B)** Over the course of SRP induction, PV+ cells showed a significant decrease in stimulus-evoked fluorescence, eventually displaying suppression below baseline levels. PV+ activity for N stimuli was also significantly increased relative to activity for *F* stimuli. **(C)** SOM+ cells showed the opposite pattern of changes to PV+ cells, with an increase in stimulus-evoked fluorescence over the course of SRP induction and a significant decrease in fluorescence in response to N stimuli relative to *F* stimuli. Δ*F*/*F* calculated as *F*_stim_ – *F*_gray_/*F*_gray_. Replotted from Hayden et al. (2021). **(D,E)** Chemogenetic suppression of PV+ cells occludes SRP. **(D)** PV-Cre mice were injected with a virus expressing a Cre-dependent inhibitory DREADD receptor (hM4Di). The mice then underwent a standard SRP protocol, and following the familiar/novel test on day 7 received a systemic injection of CNO in order to activate hM4Di receptors and suppress PV+ cell activity. 1 h later mice viewed the original familiar stimulus and a new novel stimulus. **(E)** Mice showed normal SRP expression before CNO injection, but after injecting CNO to suppress PV+ cell activity mice showed an increase in VEP magnitude which eliminated the familiar-novel difference. **(F,G)** Optogenetic activation of PV+ cells partially reverses SRP. **(F)** PV-Cre mice were injected with a virus expressing Cre-dependent ChR2 and implanted with an optic fiber above bV1. Following SRP induction, mice viewed familiar and novel stimuli with blue-light activation of PV+ cells on alternating blocks on the test day. **(G)** Mice showed normal SRP expression during blocks in which PV+ cells were not activated by blue light, but the familiar-novel difference was significantly reduced for light-on blocks, due to a reduction in VEP magnitude. Panels **(D–G)** replotted from Kaplan et al. (2016). Asterisks denote significant differences throughout while non-significant differences are shown as n.s. In the final panel, actual *p*-values are provided.

Cortical PV+ inhibitory neurons have been implicated in key aspects of visual function, including critical period OD plasticity (Hensch, 2005; Maffei et al., 2006), orientation selectivity (Atallah et al., 2012; Lee et al., 2012; Wilson et al., 2012), and visually-induced gamma oscillations (Cardin et al., 2009; Sohal et al., 2009; Carlen et al., 2012; Chen et al., 2017; McNally et al., 2020). Experimental evidence now indicates a key role for these interneurons in expression of SRP and accompanying habituation. First, cell type-specific calcium imaging within L4 of binocular V1 reveals that PV+ cells are highly responsive to novel oriented sinusoidal grating stimuli, but that this activity diminishes over the time-course of SRP and, eventually, is suppressed below baseline activity levels by highly familiar oriented stimuli (Figures 3A,B) (Hayden et al., 2021). Second, LFPs recorded within L4 across the same time-course reveals high gamma oscillations (65–85 Hz), which are a hallmark of the engagement of PV+ neurons (Cardin et al., 2009; Sohal et al., 2009; Carlen et al., 2012; Chen et al., 2017; McNally et al., 2020), during novel stimulus viewing. The power within the high gamma frequency range is reduced as the stimulus becomes progressively familiar but re-emerges upon the presentation of a new novel stimulus (Hayden et al., 2021). Third, temporary inactivation of PV+ neurons in V1 using a chemogenetic approach mimics and occludes expression of SRP (Figures 3D,E) (Kaplan et al., 2016). Fourth, activation of PV+ neurons using optogenetics inhibits V1 activity and also abrogates the selective response to familiar and novel stimuli (Figures 3F,G) (Kaplan et al., 2016). Finally, loss of NMDA receptor expression within PV+ neurons in a cell type-specific knockout mouse significantly impairs SRP and OSH expression (Kaplan et al., 2016). Thus, multiple lines of evidence implicate these interneurons as a key player within cortical familiarity/novelty detection.

Recent work has revealed that SOM+ neurons may also contribute to SRP, but through an opposing pattern of stimulus-induced activity. Calcium imaging indicates that SOM+ neurons initially show low levels of activity in response to novel stimuli, but this activity increases as stimuli become more familiar across days, following a similar time-course as SRP (Figure 3C) (Hayden et al., 2021). As with PV+ neurons, SOM+ neuronal activity can be inferred by measuring oscillatory power of the LFP or EEG. Recent experiments have shown that SOM+ cell activity generates relatively low frequency (10–30 Hz) oscillations (Chen et al., 2017; Veit et al., 2017; Hakim et al., 2018; Huang et al., 2020). These are typically characterized as alpha/beta oscillations, although some confusion has arisen from the fact that the 25–30 Hz oscillations have also been described as “gamma” by some authors (Veit et al., 2017; Hakim et al., 2018). Semantics aside, as the stimulus is repeatedly shown over days, 10–30 Hz power within L4 V1 LFP increases (Hayden et al., 2021). Again, this change is stimulus specific, as 10–30 Hz oscillatory power is substantially reduced in response to a novel stimulus relative to the familiar stimulus.

Together, these oscillatory patterns and calcium imaging results show that SOM+ inhibitory neurons are preferentially engaged while mice view familiar stimuli, and that PV+ inhibitory neurons are preferentially engaged while mice view novel stimuli. They are also consistent with other studies which have found that SOM+ cells in L2/3 increase their activity following daily exposure to an unrewarded stimulus, whereas PV+ cells in L2/3 decrease their activity (Kato et al., 2015; Makino and Komiyama, 2015). Thus, one simple model of SRP could be recruitment of a canonical interneuron circuit, wherein familiar stimuli activate SOM+ cells which in turn decrease the activity of PV+ cells and augment VEPs in L4.

One curiosity of these results is the clear stimulus-specificity of the inhibitory interneuronal responses. Inhibitory interneurons are generally thought to be comparatively unselective for orientation (Tremblay et al., 2016; Hooks and Chen, 2020), by virtue of inputs from a wide range of excitatory neurons with various orientation selectivities (Kerlin et al., 2010). However, a closer inspection of data in mouse V1 shows that, while many GABAergic neurons show no or limited orientation preference, many do have a moderate orientation preference (Kerlin et al., 2010; Ma et al., 2010; Makino and Komiyama, 2015). Learning, albeit rewarded, has also been shown to increase the stimulus selectivity of PV+ interneuron populations (Khan et al., 2018; Poort et al., 2021), and SRP experiments show that SOM+ interneurons can gain orientation selectivity simply by exposure to novel gratings of a single orientation (Hayden et al., 2021). Presumably this acquisition of selectivity reflects an increased response to a subset of excitatory inputs conveying information about the familiar stimulus. The increased postsynaptic response of SOM+ cells could be a passive reflection of increased presynaptic activity in these excitatory inputs, or it could arise from potentiation of these synapses.

As mentioned above, loss of NMDA receptors from PV+ neurons impairs expression of both SRP and OSH, raising the possibility that modifications of glutamatergic input to the PV+ neurons might mediate this learning. In considering this hypothesis, it is important to note that whereas NMDA receptors are expressed at many of the synapses impinging on PV+ neurons, there are actually very few at the direct thalamocortical synapses (Kloc and Maffei, 2014). Further, blocking NMDA receptors on PV+ neurons produces network effects revealed by major changes in cortical oscillations (Korotkova et al., 2010; Carlen et al., 2012) that can mimic those observed with SRP (Hayden et al., 2021). Indeed, acute application of the non-competitive NMDA receptor blocker ketamine, which is known to preferentially affect PV+ inhibitory interneurons due to the increased channel opening of their NMDA receptors (Homayoun and Moghaddam, 2007), also occludes the expression of well-established SRP in the VEP, similar to what is observed following direct chemogenetic inactivation of PV+ neurons (Kaplan et al., 2016). This temporary masking of familiar-novel differences in the VEP indicates that loss of NMDAR function in PV+ neurons may disrupt SRP expression rather than impairing SRP acquisition.

The study of the LFP within the SRP paradigm indicates a mutual opposition between alpha/beta oscillations and high gamma oscillations within L4 of V1 in the awake mouse (Hayden et al., 2021). In line with evidence that SOM+ neurons inhibit PV+ neurons with little reciprocal inhibition of PV+ neurons onto SOM+ neurons, it is tempting to imagine that an exploratory mode of operation within V1 that maximizes sensory fidelity involves strong feedforward engagement of PV+ neurons and increased high gamma power, as observed for novel stimulus presentations, while memory of a familiar but innocuous stimulus engages SOM+ neurons to suppress these PV+ neurons. Future work using calcium imaging to record from SOM+ and PV+ neurons combined with interventional approaches to manipulate their activity will be required to directly confirm this arrangement. It will also be important to interrogate the activity of VIP+ inhibitory interneurons given recent observations that these cells, like PV+ cells, decrease their activity once familiarized with a stimulus, regardless of whether it was through passive exposure or part of a reward learning paradigm (Makino and Komiyama, 2015; Garrett et al., 2020).

## Passive Viewing: Evidence Outside of the Standard Stimulus-Selective Response Plasticity Paradigm

Various forms of experience-dependent plasticity have been demonstrated in L2/3 of mouse sensory cortex (Kato et al., 2015; Makino and Komiyama, 2015; Fiser et al., 2016; Garrett et al., 2020; Henschke et al., 2020; Wang et al., 2020; Gao et al., 2021; Poort et al., 2021). Of particular relevance to SRP are those studies that recorded, either by design or as a control, the changes that are caused by passive experience. In general, familiarity that occurs due to the passive presentation of visual or auditory stimuli causes both principal cells and PV+ cells in L2/3 to decrease their activity compared to baseline or novel stimuli, as measured with genetically encoded calcium sensors (Kato et al., 2015; Makino and Komiyama, 2015; Gao et al., 2021). Conversely, L2/3 SOM+ cells increase their response when passively viewing a familiar stimulus (Kato et al., 2015; Makino and Komiyama, 2015). These findings can change with the presence of reward, attention, or a task (Makino and Komiyama, 2015; Fiser et al., 2016; Henschke et al., 2020; Wang et al., 2020; Poort et al., 2021). These observations in L2/3 fit well with calcium imaging in V1 L4 for principal, PV+ and SOM+ neurons (Kim et al., 2020; Hayden et al., 2021). Thus, the signatures for stimulus familiarity using calcium imaging appear to be similar in granular and supragranular layers in both auditory and visual cortex.

It is tempting to speculate that familiarity may have similar effects regardless of cortical region or species. In evaluating this hypothesis, we must rely on unit spiking activity from neurons whose type (transcriptome) may not be known. To briefly review passive experience-dependent plasticity in V1, calcium imaging of principal cells in L4 show reduced *average* activity during a block of familiar stimulus viewing (Kim et al., 2020) and patch-clamp recordings of L2/3 neurons under have shown a reduction in *average* activity (Gao et al., 2021). We have also shown L4 single-units have an increase in the *peak* rate of action potential firing that is elicited by each phase reversal of the visual stimulus, corresponding to the potentiated VEP response (Cooke et al., 2015). As will be discussed further below, and already mentioned above, these observations can be reconciled if there is a reallocation of when spikes occur during familiar stimulus viewing. However, in the meantime we can now consider the hypothesis that the fundamental process of familiarity encoding, measured as a reduction in the *average* firing rate and an increase in the *peak* firing rate, is shared across areas of cortex and species.

In seminal studies using macaque monkeys, it has been shown that sets of familiar stimuli cause less *average* activity in regular-spiking units in higher-order visual or polymodal regions of cortex, including inferotemporal (IT), perirhinal, and entorhinal cortex (Li et al., 1993; Xiang and Brown, 1998; Rainer and Miller, 2000; Freedman et al., 2006; Anderson et al., 2008; Woloszyn and Sheinberg, 2012; but see Holscher et al., 2003). Similarly, in rat perirhinal cortex, reduced neural activity in response to familiar stimuli has also been observed (von Linstow, Roloff et al., 2016). These findings are in agreement with what is observed with unit recordings in superficial layers (Gao et al., 2021) and our own calcium imaging data in L4 (Kim et al., 2020). Namely, there is diminished *average* cellular activity to familiar stimuli. However, the question remains: is there evidence from prior studies that the *peak* firing rate to familiar stimuli is increased, as we have observed? (Cooke et al., 2015).

In considering this question, it is important to call attention to some key methodological differences. In the monkey studies, the relative response rates, or “tuning curves,” are generated by collecting spikes in response to a collection of pictures of randomly-selected objects or faces. A familiar tuning curve is thus the responses of a unit to this collection of familiar images. A novel tuning curve is acquired similarly, but using a set of images the animal has never seen. Defining the relative response rate in these studies is challenging and often authors only used very few images (∼10) to define the tuning curve. The most thorough study in macaque area IT was performed by Woloszyn and Sheinberg (2012), who used 125 images for their familiar or novel stimulus sets (Woloszyn and Sheinberg, 2012). When the activity of each unit was averaged across the complete set of images, the data again showed an overall decrease in firing when monkeys viewed the familiar stimuli compared to novel, replicating previous findings. However, when they asked how the *peak* firing to the *one best stimulus* in each set varied with familiarity, they observed a greater response to the familiar than to the novel stimulus, consistent with our findings in L4 of mouse V1. Woloszyn and Sheinberg (2012) propose a model wherein familiarization sharpens selectivity of excitatory cells, causing them to fire maximally (beyond even the most preferred novel stimulus) to a few preferred images and minimally (below even the least-preferred novel stimulus) to non-preferred images. Evidence supporting this hypothesis could be seen in early studies, even if the authors failed to construct an exhaustive tuning curve, so long as they looked at selectivity. Indeed, despite the reduction in firing to the average familiar stimulus within the limited range of stimuli presented, familiar tuning curves are often more selective for a given familiar stimulus compared to a novel tuning curve (Rainer and Miller, 2000; Freedman et al., 2006; Anderson et al., 2008; Woloszyn and Sheinberg, 2012).

In the context of mouse V1 L2/3, the analogous modification would be an increase in orientation or direction selectivity, both of which have been shown to occur with passive experience, despite the reduction in average firing rate (Gao et al., 2021). At a population level, such selectivity increases sparseness and could make a group of familiar images have more orthogonal population rate vectors compared to novel images, making it easier to parse stimuli (Failor et al., 2021). Such orthogonalization could explain the benefits of familiarity on behavior. While familiar stimuli confer no clear behavioral benefit to simple tasks (D’Amato and O’Neill, 1971), more difficult tasks are more easily performed with familiar stimuli (Rainer and Miller, 2000; Rainer et al., 2004; Mruczek and Sheinberg, 2007).

Thus far, the results from mice, rats, and primates in various cortical areas and layers are consistent with respect to excitatory neurons. Is this also true for inhibitory interneurons? Fast-spiking IT neurons, which are likely PV+ cells based on their waveform and firing rate, are less active when viewing familiar stimuli, consistent with what we observe in mouse V1 using calcium imaging of genetically tagged PV+ neurons (Woloszyn and Sheinberg, 2012; Hayden et al., 2021). Additionally, these neurons display quick onset and offset dynamics, ostensibly to help with stimulus processing (Meyer et al., 2014). It is difficult to determine SOM+ cell activity in primates due to a lack of genetic targeting methods and less obvious electrophysiological signatures in this cell type. Thus, the question of whether or not SOM+ cells in primate IT increase their activity with familiarity will likely remain unanswered in primates until further methodology is developed. Regardless, a common motif in primary sensory areas of mice and in higher-order visual cortex of primates is that familiarity reduces the *average* population activity of both PYR and PV+ cells while increasing the *peak* activity to the neuron’s preferred familiar stimulus.

## Differences in Methods Used to Record Stimulus-Selective Response Plasticity and Accompanying Habituation

Much of our understanding of SRP and accompanying learning effects has come from measuring the magnitude of VEPs. As with many other techniques (e.g., intrinsic signal optical imaging), VEPs are a somewhat indirect measure of visual processing, reflecting the flow of positive current into radially aligned dendrites within V1 (Mitzdorf, 1985; Kirkwood and Bear, 1994; Aizenman et al., 1996), but they provide real-time resolution for electrical events occurring within the cortex. While the number of cells and synapses contributing to the VEP is not nearly as large as that recorded with the closely related signal of EEG (Katzner et al., 2009), it remains a population signal and there are benefits and limitations to its use. Changes in the trough-peak magnitude of VEPs as a result of sensory deprivation or supplementary experience can provide a wealth of information about the boundary conditions of visual cortical plasticity and, to some extent, underlying mechanism when combined with interventional approaches (Porciatti et al., 1999; Sawtell et al., 2003; Frenkel and Bear, 2004; Frenkel et al., 2006). Recordings are stable and well suited to longitudinal measurements of plasticity over days, and there is great opportunity for translation into humans using EEG recordings given the extensive use of VEPs in the clinical setting (Lascano et al., 2017). The recent observations of shifts in the frequency composition of L4 LFP during SRP (Hayden et al., 2021) only increase the optimism for this translational approach, although it is important to note that great care must be taken when trying to measure VEPs and oscillations in the same experiments, given the non-stationarity of the signal acquired after phase reversals (Cohen, 2017). Further work will be required to determine the extent to which the LFP phenomenology of either type is accessible with the use of surface EEG recordings in mice or in humans.

Clear limitations exist with the VEP methodology and major gaps remain in our understanding of SRP as a result. Single-synapse functional and structural resolution will eventually be required to develop a more complete understanding of SRP. However, some insight has recently been provided at single cellular resolution. As discussed above, electrophysiological recordings of action potentials from individual neurons in L4 indicates that the maximal firing rate after stimulus phase reversal is greater for familiar than for novel stimuli Temporally, this peak firing rate coincides with the early trough (peak negativity) of the VEP, which is dramatically potentiated in SRP (Frenkel et al., 2006). Interestingly, however, a quiescence of firing is apparent after this peak in response to familiar stimuli that does not occur for novel stimuli, suggesting that spikes have been re-distributed across the 500-ms time window that occurs between stimulus phase reversals under a standard SRP protocol (Cooke et al., 2015). The temporal profile of firing rates evoked by familiar and novel stimuli needs to be investigated further, but we suggest it is perhaps too simple to say that firing rate is increased (or decreased) by stimulus familiarity without taking time from stimulus onset into consideration.

Such a redistribution of spikes elicited by phase reversals of familiar stimuli might explain the apparent discrepancy between the electrophysiological observation of increased peak firing, and the results using calcium imaging of L4 principal neurons showing reduced activity during blocks of stimulation (Makino and Komiyama, 2015; Kim et al., 2020). To briefly review, the genetically encoded calcium sensor GCaMP6f was expressed within L4 excitatory neurons using a specific Cre recombinase line, and the signal in these L4 neurons was found to be significantly greater for novel stimuli than familiar stimuli, in line with other calcium imaging studies of habituation effects in L2/3 of primary sensory cortex (Kato et al., 2015; Makino and Komiyama, 2015). Interestingly, the calcium signal measured from L4 in the neuropil, which likely reflects dendritic calcium, showed the reverse pattern of selectivity, with greater stimulus-evoked fluorescence for familiar stimuli relative to novel stimuli, in line with what is observed for the VEP (Kim et al., 2020). Nevertheless, the calcium imaging data is clearly at odds with previous single unit electrophysiological observations (Aton et al., 2014; Cooke et al., 2015). While deconvolution methods can be applied to calcium imaging experiment to infer single spike resolution (Pachitariu et al., 2018), typical imaging experiments are biased toward detecting bursts of action potentials over individual action potentials (Huang et al., 2021), making direct comparisons between calcium imaging and electrophysiological experiments difficult.

## Putting the Pieces Together

While a great deal of progress has been made, the complexity of the cortical microcircuit is such that there remain many possibilities for how that circuitry supports many of the features of SRP. In the canonical cortical microcircuit, inputs from the thalamus target principal cells in L4, which subsequently transmit information to L2/3 and then L5 and L6. However, feedback and direct connections between these layers, as well as the complexities of interneuronal connections, allow many viable paths for learning and memory to modulate.

The question that then faces us is how the cortical circuit can support the many aspects of SRP that have been described over the years. There are a number features of SRP that must be accounted for in any potential circuit model: (1) SRP requires NMDA receptor expression in V1, but not in L4 principal cells; (2) SRP exhibits stimulus- and eye-specificity; (3) SRP is observed in L4, both as an increase in VEP magnitude and peak firing rate but as a decrease in calcium flux into L4 principal cell somata; (4) SRP expression correlates with and may be driven by an increase in SOM+ cell activity, and a decrease in PV+ cell activity; (5) SRP is not apparent at the onset of a block of familiar stimuli, but requires some intracortical activity to emerge during phase reversals, possibly including thalamic feedback; (6) expression of SRP requires some period of offline consolidation (Aton et al., 2014); (7) SRP is mimicked and occluded by LTP-like effects on thalamocortical transmission resulting from high-frequency thalamic stimulation. Now we describe a number of potential models for how the circuitry of V1 might support SRP.

### Possibility 1: Stimulus-Selective Response Plasticity Is Long-Term Potentiation of Thalamocortical Synapses in L4

**FIGURE 4:**
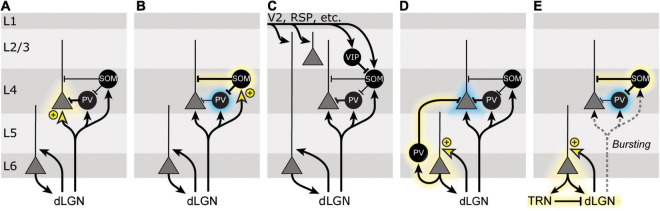
Models of SRP. **(A)** Neurons in the dorsal lateral geniculate nucleus (dLGN) project to several populations of neurons in V1, including excitatory principal cells (gray triangles) and inhibitory interneurons (black circles) in multiple layers. SRP was originally interpreted as potentiation of a subset of thalamic inputs onto L4 principal cells (indicated by yellow arrowhead and + symbol), which would lead to an increased response of L4 principal cells (indicated by yellow halo) for familiar stimuli. The bulk of the evidence argues against this simple model of SRP. **(B)** Following SRP induction, somatostatin-positive (SOM+) inhibitory interneurons show heightened activity for familiar stimuli, and parvalbumin-positive (PV+) inhibitory interneurons show reduced activity for familiar stimuli (indicated by blue halo). This could be mediated by the potentiation of thalamic inputs onto L4 SOM+ cells, which inhibit L4 PV+ cells. **(C)** V1 receives a great deal of top-down feedback from other cortical and subcortical areas, including V2, retrosplenial cortex (RSP), and the basal forebrain. Changes in the feedback from these brain regions might explain some features of SRP, though how this model would also incorporate the necessity of local plasticity in V1 is unclear. **(D)** L6 corticothalamic cells also receive substantial thalamic input and have been implicated in SRP. Potentiation of thalamic inputs onto L6 principal cells might alter L4 activity via a population of PV+ cells known to inhibit other cortical layers. **(E)** Alternatively, L6 corticothalamic neurons could shift neurons in the dLGN from a tonic firing to a burst firing mode, via feedback to the thalamic reticular nucleus (TRN). This change in firing patterns could then preferentially recruit L4 SOM+ cells over PV+ cells to alter ongoing activity in L4.

The simplest model which was originally used to explain SRP was that it is a manifestation of thalamocortical Hebbian synaptic potentiation onto L4 principal cells (Figure 4A). While this hypothesis is appealing due to its simplicity and the fact that it can account for eye-specificity of SRP, it fails to account for many of the other features of SRP, chief among them that it is unaffected by knocking out NMDA receptors in L4 principal cells (Fong et al., 2020). Additional strong evidence ruling out this possibility arises from the observation that pharmacological isolation of thalamocortical synapses in L4 eliminates SRP expression (Cooke and Bear, 2014) and from the gradual emergence of SRP with repeated stimulus presentations.

### Possibility 2: Stimulus-Selective Response Plasticity Reflects Plasticity Onto Inhibitory Interneurons

The recent findings that SRP expression correlates with an increase in SOM+ cell activity and depends on a decrease in PV+ cell activity highlights the importance of interneurons in this phenomenon. It is therefore tempting to speculate that changes in the strength of synapses onto one of these populations of interneurons is responsible for SRP (Figure 4B). In particular, PV+ interneurons receive dense thalamocortical input (Cruikshank et al., 2007; Ji et al., 2016) and knocking out NMDA receptors from PV+ cells in V1 disrupts SRP expression (Kaplan et al., 2016), consistent with the possibility that Hebbian synaptic depression of thalamocortical synapses onto PV+ cells in L4 might account for SRP. Data that argue against this hypothesis include the disruption of SRP by molecular manipulations that are selective for LTP (Frenkel et al., 2006; Cooke and Bear, 2010), and evidence that thalamocortical inputs to PV+ cells within L4 of V1 lack NMDA receptors (Kloc and Maffei, 2014), ruling them out as the locus of NMDAR-dependent plasticity.

Alternatively, SOM+ inhibitory neurons are well situated to modulate PV+ neurons and L4 principal cells, and potentiation of thalamocortical inputs onto this cell population could explain various features of SRP. However, these cells receive relatively few thalamic inputs (Tremblay et al., 2016) and, at least within sensorimotor cortex of mice, Hebbian synaptic plasticity at excitatory synapses onto SOM+ cells is not NMDAR-dependent (Chen et al., 2009). Further experiments will be required to fully rule out this possibility.

### Possibility 3: Stimulus-Selective Response Plasticity Involves Top-Down or Interlaminar Feedback

Although SRP is clearly disrupted by manipulations local to V1, we cannot rule out the possibility that top-down feedback from other cortical areas may play a role in SRP (Figure 4C). Indeed, other groups have found that inputs from higher-order visual areas exhibit precise control over inhibition within V1, and strongly influence how V1 processes even simple visual stimuli. For example, feedback from cingulate cortex plays an important role in shaping the responses of superficial neurons in V1 to visual stimuli (Zhang et al., 2014), and feedback from motor areas communicating motor information exerts strong control over the activity of different populations of inhibitory neurons in V1 to modify visual cortical processing (Kaneko et al., 2017; Pakan et al., 2018). A strong candidate might be retrosplenial cortex, identified as exerting influence on V1 during an associative learning task (Makino and Komiyama, 2015). Some computational models have even posited rewarded experience-dependent changes in L2/3 of V1 as a combination of the standard interneuron circuit and VIP+ cells receiving top-down input (Wilmes and Clopath, 2019). However, this route of influence is less likely for passive viewing, as unrewarded familiarity does not change retrosplenial cortical axonal activity (Makino and Komiyama, 2015). Another interesting possibility involves cholinergic projections from the basal forebrain. Activation of acetylcholine axons from the basal forebrain increases power in high frequencies whereas deactivation increases power in low frequencies (Goard and Dan, 2009; Pinto et al., 2013). Thus, SRP could be explained via engagement of a cholinergic signal, although global changes linked to general arousal are unlikely (Hayden et al., 2021).

Perhaps another visual cortical region provides feedback information. L2/3 cells receive feedback from V2 upon their dendrites near the L1 border, consistent with the idea that dendritic connections usually originate from top-down sources and peri-somatic connections usually originate from bottom-up sources within this cortical layer (Gillon et al., 2021; Young et al., 2021). In fact, novel object recognition is eliminated in rats if L6 cells in V2 are selectively ablated (Lopez-Aranda et al., 2009). Although this experiment did not discriminate between corticothalamic and corticocortical L6 cells, this surprising experimental result may provide important insight. We will revisit the potential role of L6 neurons below.

It is also important to note that feedback may arise more locally from within V1, which would explain the efficacy of blocking NMDAR function local to V1. It may be that L5 excitatory neurons strengthen their connection onto L2/3 excitatory neurons within V1, providing a feedback signal to convey familiarity. Such intracortical strengthening has indeed been demonstrated in mouse V1 after passive visual experience (Gao et al., 2021). Since this signal is excitatory, one would assume L2/3 cells should fire more. However, it has also been shown that familiarity reduces the strength of thalamocortical synapses upon L2/3 cells (Gao et al., 2021). In addition, the L5 regular-spiking to L4 fast-spiking connection is strengthened with experience, perhaps inhibiting L4 feedforward information to L2/3 and thus explaining the reduction of activity in L2/3 cells to familiar stimuli (Kissinger et al., 2020). However, our data and that of others has shown that the firing rate in L4 and deeper layers for familiar stimuli is larger than for novel (Aton et al., 2014; Cooke et al., 2015; Durkin et al., 2017). Further complicating matters is the finding that L2/3 cell can have both positive and negative mismatched effects when comparing the effects of expectation and visual flow (Jordan and Keller, 2020). This finding highlights the striking role that local interneurons can play in inverting the polarity of a signal.

### Possibility 4: Stimulus-Selective Response Plasticity Involves Layer 6 Corticothalamic Neurons

A fourth and particularly interesting possibility for SRP comes from L6 CT cells. In mouse V1, L6 cortico-thalamic (CT) cells exhibit ultrasparse firing, are exquisitely tuned to orientation and direction, and have many top-down connections from retrosplenial cortex and V2 (Velez-Fort et al., 2014). Purely cortico-cortical (CC) L6 neurons, on the other hand, are more broadly tuned and have very few long-range connections (Velez-Fort et al., 2014). Thus, L6 CT cells are well-positioned to respond selectively to familiar stimuli and may even do so via top-down connections.

About 65% of all excitatory cells in L6 are CT cells and can be selectively manipulated using the Neurotensin Receptor 1 (Ntsr1)-Cre mouse line (Olsen et al., 2012; Bortone et al., 2014; Velez-Fort et al., 2014). Surprisingly, these L6 CT cells are also connected to unusual translaminar projecting PV+ cells within L6 (Bortone et al., 2014), as well as PV+ neurons in L4 (Kim et al., 2014), either of which enable CT L6 neurons to inhibit L4 neurons via a purely intracortical route. Thus, L6 CT cells are excellent candidates for exerting an inhibitory influence on L4 neurons during SRP (Figure 4D). Studies have indeed shown that optogenetic manipulation of Ntsr1-Cre+ cells affects sound detection and discrimination across layers as well as behavior (Guo et al., 2017; Voigts et al., 2020). In fact, L6 CT cells have been implicated in passive stimulus-selective response plasticity already. It has been shown that sleep deprivation disrupts experience-dependent plasticity (Aton et al., 2014), and in a follow-up experiment, these investigators showed that experience-dependent plasticity is disrupted when L6 CT cells are inhibited specifically during NREM sleep (Durkin et al., 2017). While these investigations did not specifically study the translaminar L6 PV+ cells, they did show that fast-spiking infragranular cells increase their activity with familiarity (Aton et al., 2014). Further experiments elucidating whether superficial and L4 PV cells of the canonical interneuron circuit have different responses to familiarity than the special L6 translaminar PV+ cells will be of great interest. Regardless, these papers collectively show that L6 CT cells influence the cortical column via intracortical influences on PV+ cell activity and that L6 CT cells are an integral part of SRP during NREM sleep. However, it is important to note that L6 CT cells obviously provide substantial excitation to the thalamus as well.

As mentioned, SRP is only apparent at the first phase reversal of the familiar grating, not at stimulus onset. This delay gives ample time for recruitment of both direct and indirect circuits by which an LTP-like process in V1 principal cells could lead to increased SOM+ cell activation. Indeed, more immediate and shorter-term changes in firing to the familiar stimulus are observed in the dLGN during the first recording session (Durkin et al., 2017). An intriguing possibility is that corticothalamic feedback may serve to initiate SRP by switching dLGN firing from tonic to burst firing mode in response to familiar stimuli. Thalamic bursting occurs when membrane hyperpolarization rebounds and relies on the interplay of hyperpolarization-induced cation currents (Ih) and voltage gated calcium channels (Steriade and Llinas, 1988; McCormick and Feeser, 1990) [primarily the CaV3.1 channel in dLGN and CaV3.3 in the thalamic reticular nucleus (TRN)] (Steriade and Llinas, 1988; McCormick and Feeser, 1990; Talley et al., 1999; Kim et al., 2001). Burst firing could contribute to VEP potentiation both by increasing coincident spiking input to cells in V1, and by recruiting the activity of SOM+ neurons. Relative to PV+ cells, SOM+ cells receive sparse TC innervation (Tremblay et al., 2016) but exhibit disproportionate activation by burst versus tonic spikes (Hu and Agmon, 2016). Thus, a mode shift of dLGN firing could trigger a switch in the activity of V1 inhibitory networks that manifests as SRP (Figure 4E). Future experiments that elucidate which, if any, of these possible circuits are responsible for SRP will be of great interest.

## Summary

Much progress has been made over the last few years to understand the mechanisms and significance of SRP. There are now several new lines of evidence that have provided substantial insight into its molecular and circuit-level implementation, as well as strong evidence for its role in long-term habituation. Clearly, SRP is not a simple phenomenon supported only by feedforward Hebbian synaptic plasticity, despite the requirement for key Hebbian molecular mechanisms. It is increasingly apparent that SRP also requires the engagement of the now well-documented SOM+–PV+ disinhibitory circuit, as well as some form of feedback circuit that triggers the retrieval of long-lasting familiarity, which in turn enables novelty detection. In addition, sleep plays a key role in the consolidation of familiarity that only manifests as potentiation after several hours. Key questions that remain to be answered are whether SRP relies purely on events occurring within V1 or whether there is a critical influence from other structures, either through top-down input from other cortical regions, or by recruiting a corticothalamic loop to influence the activity of L4. While great insight has been provided into the key role for different cell types and cortical layers by recording the activity of select cell types and intervening with cell type-specific knockdowns and chemo/optogenetics, questions remain over why different methods for recording cortical neuronal responses yield mismatched readouts of SRP. On the one-hand, electrophysiological recordings of LFP and unit activity reveal a substantial potentiation of peak phasic cortical response to familiar stimuli, while on the other calcium imaging reveals a diminished response. Further work will be required to understand this discrepancy. Finally, questions remain about the translational utility of SRP. The indications are that SRP may be dysregulated in disorders of the brain that affect learning and memory, whether in development, sleep, aging or dementia. Recording SRP-like effects on event-related potentials and oscillations using non-invasive EEG in human subjects may present an opportunity for diagnosis, patient stratification, and assessment of response to treatment. The utility of this approach will only be aided by continuing to work on the phenomenon in the mouse to further develop our burgeoning understanding of underlying mechanism at a molecular, circuit and systems-level.

## Author Contributions

DM, DH, FC, SC, and MB contributed to the manuscript. DM and FC prepared the figures. All authors contributed to the article and approved the submitted version.

## Conflict of Interest

The authors declare that the research was conducted in the absence of any commercial or financial relationships that could be construed as a potential conflict of interest.

## Publisher’s Note

All claims expressed in this article are solely those of the authors and do not necessarily represent those of their affiliated organizations, or those of the publisher, the editors and the reviewers. Any product that may be evaluated in this article, or claim that may be made by its manufacturer, is not guaranteed or endorsed by the publisher.
